# Waves of Endurance: A Pilot Study of Beta Brain Wave Signatures Linked to Immune Adaptation in Transatlantic Rowers

**DOI:** 10.7759/cureus.84059

**Published:** 2025-05-13

**Authors:** Merin Chandanathil, Daniel P Longman, Tomasz Nowak, Jonathan C.K. Wells, Jay T Stock, Michael P Muehlenbein, Hannah Schneiders, Nicola L Kelly, Vasavi R Gorantla, Courtney C Lewis, Richard M Millis

**Affiliations:** 1 Physiology, American University of Antigua, St. Johns, ATG; 2 School of Sport, Exercise and Health Sciences, Loughborough University, Loughborough, GBR; 3 Anthropology, Baylor University, Waco, USA; 4 Childhood Nutrition Research Centre, UCL (University College London) Institute of Child Health, London, GBR; 5 Anthropology, University of Western Ontario, London, CAN; 6 Department of Anthropology, Baylor University, Waco, USA; 7 Anesthesiology, Southeast Scotland School of Anaesthesia, Edinburgh, GBR; 8 Medicine, University of Exeter and Torbay and South Devon NHS Foundation Trust, Torquay, GBR; 9 Medical Education, California University of Science and Medicine, Colton, USA; 10 Clinical Medicine, American University of Antigua, St. Johns, ATG

**Keywords:** body weight loss, hemolytic complement activity, immunological association, leptin association, quantitative electroencephalography, ultra-endurance sports

## Abstract

Background: Ultra-endurance athletics, such as transoceanic rowing, imposes significant physiological stress, leading to muscle catabolism and alterations in immune function. A case series pilot study from our laboratory suggests that the central nervous system may mirror these changes through a pattern of disproportionately high beta brainwave voltage amplitude activity that promotes vigilance. This study investigates the relationship between quantitative electroencephalographic (qEEG) brainwave patterns and markers of innate immune responses in a group of transatlantic rowers post-competition.

Methods: Twenty-four transatlantic rowers (18 male, 6 female; mean age 31 ± 11 years) were assessed immediately after completing a transatlantic rowing competition of 30 to 53 days duration. EEG recordings measured voltage amplitudes of slow delta (1-3 Hz), theta (4-7 Hz), alpha (8-12 Hz), and fast beta (13-30 Hz) brainwaves under eyes-open and eyes-closed conditions. Pre- and post-race plasma cortisol, testosterone, leptin, myoglobin, total antioxidant content (TAC), malondialdehyde (MDH), collagen oligomeric matrix protein (COMP), IL-6, bacterial killing activity (BKA), and hemolytic complement activity (HCA) assays were used to assess changes in stress responses and immune functions. Student’s paired t-test and Pearson’s product-moment coefficient were used to evaluate the significance of pre- versus post-race differences in oxidative stress- and immunologic-related biomarkers, as well as correlations between the post-race qEEG parameters and the pre- versus post-race biomarker differences.

Results: The changes in body weight were significantly negatively correlated with the changes in cortisol and COMP (r = -0.40, p < 0.05) and significantly positively correlated with the changes in leptin (r = +0.6, p < 0.01). Voltage amplitudes of fast beta waves, under both eyes-open and eyes-closed conditions, positively correlated with the pre- versus post-race percent change in BKA (r = +0.42, +0.44, p < 0.05) and negatively correlated with the pre- versus post-race percent change in HCA (r = -0.65, -0.66, p < 0.01).

Conclusion: These findings support the hypothesis that prolonged intense physical exertion may induce a pattern of intense cerebral cortical activation correlated with immune modulation. The increased fast beta activity positively correlated with enhanced bacterial killing and decreased HCA, suggesting a link between cortical arousal and immune adaptation. These findings underscore the interconnectedness of neurophysiological states and physiological stress responses in ultra-endurance athletes.

## Introduction

Ultra-endurance athletics, such as transoceanic rowing, represents profound physical and psychological challenges, pushing human adaptive mechanisms to their limits. The average age of participants in transoceanic ocean rowing events, such as the Talisker Atlantic Challenge, is approximately 35 to 40 years, with a wide range from the early 20s to late 60s. The field has historically been predominantly male, although female participation is steadily increasing. The Atlantic crossing typically takes between 30 and 70 days, depending on weather conditions and crew size. Participation has grown in recent years, with around 30 to 40 teams (including solo rowers and crews of two to five people) completing the crossing annually. This demographic profile is broadly consistent with that of ultra-endurance athletes more generally [1]. These endeavors involve a combination of extreme physical exertion, sleep deprivation, environmental unpredictability, and caloric deficits, all of which stress the human body’s metabolic and cognitive systems. The physiological impact of such activities has been studied in various forms, including metabolic shifts, hormonal changes, and neurophysiological adaptations [2-7].

Prolonged endurance activities necessitate substantial energy expenditure, prompting the body to adapt its energy utilization strategies. Initially, glycogen stores serve as the primary energy source. As these deplete, the body increasingly relies on fat oxidation to meet energy demands. This metabolic shift enhances the efficiency of energy production during extended exercise periods. However, the prolonged reliance on fat metabolism can lead to increased production of free radicals, potentially causing oxidative stress and muscle fatigue [8].

Extended endurance exercise also influences hormonal balance, notably affecting cortisol and testosterone levels. Cortisol, a stress hormone, may rise in some athletes during, but in the majority of athletes after, prolonged high-intensity exercise [9]. Elevated cortisol levels over extended periods of high-intensity exercise may suppress immune function and impair recovery. Conversely, testosterone levels may decline with prolonged endurance training, which can impact muscle maintenance and overall physical performance.

In parallel, the central nervous system (CNS) undergoes significant adaptations during ultra-endurance athletic events. Athletes often experience central fatigue, characterized by diminished neural drive to muscles, leading to decreased performance. Neurotransmitter imbalances, particularly involving serotonin and dopamine, are implicated in this process. Elevated serotonin levels can increase perceptions of effort and fatigue [10], whereas reduced dopamine levels may decrease motivation and endurance capacity [11]. Additionally, prolonged exercise can impair cerebral autoregulation, affecting blood flow to the brain and potentially leading to cognitive impairments [12]. Electroencephalographic (EEG) brainwaves and cerebral blood flow both arise from neural activity: EEG from electrical signaling and blood flow from metabolic demand. They are not the same, but they are functionally linked: when neurons are active, they generate electrical patterns and also require more oxygen and nutrients, which increases local blood flow [13].

The CNS plays a crucial role in managing these stressors, reallocating energy to maintain vigilance and decision-making capabilities under extreme conditions. This appears to be reflected in EEG brainwave patterns, including increased beta activity and altered theta-to-beta ratios (TBR), which may support energy-efficient cognitive processing and heightened vigilance [14]. Our pilot study suggests a role of stress-related hormones, such as cortisol and testosterone, in modulating these patterns, linking neurophysiological changes with systemic adaptations [14]. The present study expands upon our pilot study by analyzing a larger cohort of rowers to examine the relationship between EEG activity, markers of muscle catabolism, and immune function. Specifically, we evaluated how post-competition quantitative EEG (qEEG) brainwave patterns correlate with changes in bacterial killing activity (BKA) and in hemolytic complement activity (HCA), markers of the innate immune response, as well as biomarkers for responsiveness to physiologic, oxidative, and immunologic stressors.

## Materials and methods

Objectives

The main objective of this study was to provide a preliminary correlational analysis based on ultra-endurance post-race qEEG data to determine whether there are significant associations between qEEG and metabolic measures of stress.

Participants

The participants were rowers competing in the transatlantic rowing competition sponsored by the Talisker Atlantic Challenge, which departed San Sebastian de La Gomera (Canary Islands) on December 14, 2017, and finished at Nelson’s Dockyard in Antigua and Barbuda (Lesser Antilles Islands, West Indies) on January 13, 2018 (30 days), for the first-place winners and on the 23 days between January 13 and February 5, 2018, for the other participants. All participants completed a total distance of approximately 3,000 miles.

Biomarkers

Venous blood was drawn from the median cubital veins of the participants within two days of departure from La Gomera and within one hour of arriving at the finish line of the Atlantic Challenge at Nelson’s Dockyard, Antigua and Barbuda. Biochemical markers, indicative of various physiological stressors which may directly or indirectly affect immune functions, were measured at the start and finish lines of the race, at an interval of approximately 30 to 53 days. These markers included the cortisol hormonal generalized stress marker, the leptin hormonal (adipokine) energy balance stress marker, the testosterone sex hormonal stress marker, the collagen oligomeric matrix protein (COMP) musculoskeletal stress marker, the myoglobin muscle breakdown marker, the total antioxidant activity marker (TAC), and the malondialdehyde (MDA) oxidative stress marker, as well as the interleukin-6 (IL-6), the BKA, and hemolytic complement activity (HCA) innate immune response biomarkers. All laboratory assays were performed on de-identified specimens by personnel blinded to whether the samples were collected before or after the race.

We used a BKA assay as a functional test of the responsiveness of the innate immune system. This assay assessed the combined ability of various innate immune components, including complement proteins, antimicrobial peptides, and phagocytic cells such as neutrophils and macrophages, to eliminate pathogens through direct bacterial lysis or inhibition of bacterial growth. By exposing bacterial cultures to serum or whole blood from the test subject and quantifying the reduction in bacterial viability, the assay provided a dynamic and integrative assessment of innate immune function. High bacterial killing efficiency reflects robust immune responsiveness, while impaired killing capacity may indicate deficiencies in complement activation, phagocytosis, or antimicrobial protein production. The assay is particularly valuable in studying immune responses under conditions of infection, immunodeficiency, or stress. For example, post-exercise impairment in BKA has been associated with reduced neutrophil phagocytic activity in elite cyclists [15], in elite swimmers, skiers, wrestlers, and boxers [16], as well as in individuals with severe bacterial infections [17, 18]. The BKA protocol used serum diluted 1:12 in L-glutamine-supplemented CO₂-Independent Media (Gibco #18045). A single lyophilized E. coli pellet (MicroBiologics Epower Microorganisms #0483E7) was reconstituted in sterile phosphate-buffered saline and then diluted into a working solution, which produced approximately 200 to 300 colonies per 20 μL of aliquot. Aliquots of bacterial working solution were added to diluted serum in a microcentrifuge tube, vortexed, and incubated for 30 minutes. After incubation, the samples were spread on trypticase soy agar plates (BD BBL #211043) in triplicate and incubated overnight at 37°C. The number of colonies on each plate the next day was counted, and the percentage of bacteria killed for each sample relative to a positive control (media and bacteria only) was calculated.

The HCA measures functional activity of the complement system, a key component of the innate immune response. This assay evaluates the ability of serum complement proteins to lyse red blood cells that are sensitized with specific antibodies, simulating the classical pathway of complement activation. The extent of hemolysis is directly proportional to the integrity and responsiveness of the complement system. A robust HCA result indicates efficient activation and function of complement components, critical for pathogen opsonization, inflammation modulation, and direct microbial lysis. Conversely, reduced hemolytic activity may signify deficiencies or dysfunction in complement proteins, which are associated with increased susceptibility to infections and autoimmune disorders. The HCA is a reliable method to assess the innate immune system's capacity to respond to pathogens and is particularly useful in diagnosing complement deficiencies and monitoring immune function in conditions such as systemic lupus erythematosus and recurrent bacterial infections [19].

Quantitative electroencephalography

qEEG measurements were made while participants were seated upright using a standard electrode cap containing 19 recording electrodes at positions based on the standard 10-20 system attached to a left ear reference electrode clip. qEEG recordings were performed with a computer-based system (Brain Master, Model Discovery 20, Bedford, Ohio) under eyes-open (EO) and eyes-closed (EC) conditions for five minutes each. Artifacts were removed by visual recognition and with the aid of an online qEEG editing system (New Mind Technologies Inc., Roswell, Georgia). The qEEG data were based on artifact-free qEEG recordings, which varied from two to three minutes in duration. Magnitudes of qEEG voltage, dominant (mode) frequencies, and interhemispheric and intrahemispheric coherences were analyzed in the standard frequency bandwidth ranges of delta (1-3 Hz), theta (4-7 Hz), alpha (8-12 Hz), and beta (13-30 Hz). Artifact identification and rejection were based on a standardized artifact rejection algorithm known as S.A.R.A. (Standardized Artifact Rejection Algorithm) to automatically process and clean raw EEG data uploaded in EDF format. Following the automated procedure, we visually inspected and removed remaining artifacts manually. Such artifacts were identified as EEG signal changes that masked or disrupted the rhythmic EEG in all of the recorded channels simultaneously and included rapid upward deflections in the EEG signal, typically resulting from eye blink or horizontal eye movements; low-frequency changes, typically resulting from head movements or electrode shifts, that were identified as slow drifts in the signal; and high-frequency changes, typically resulting from muscle tension or other electrical noise [20]. Once artifacts were detected, the affected segments were either automatically or manually marked and excluded from the analysis, and the cleaned EEG data were converted to an EDF-formatted file for automated qEEG processing.

Statistical analysis

qEEG voltage amplitudes, dominant (mode) frequencies, and coherences, in each bandwidth, were averaged for the 19 electrode recording sites sampled, in accordance with the international standard 10-20 electrode placement system. Ratios of change were computed using the formula (Postrace − Prerace)/Postrace values, with post-race values serving as the reference point. This approach was chosen to align all comparative metrics with the time point at which qEEG voltage amplitude data were collected, immediately after the race. Post-race values were used as the denominator to standardize comparisons across subjects by minimizing the impact of inter-individual variability in baseline status [21, 22]. The Pearson product-moment coefficient was computed to determine the correlation between the pre- versus post-race ratios of change in BKA or HCA and the voltage amplitudes, dominant frequencies, and coherences in each bandwidth (Excel). The Pearson correlation coefficient table of critical values was used to determine whether the correlations were significant. Correlation coefficients of r < +0.4 or r < -0.4, R² < 0.16, p > 0.05 were not significant; r > +0.4 or r > -0.4, R² > 0.16 were reported as significant at p < 0.05 (N = 21, 19 degrees of freedom). Statistical power computations were based on Cohen’s effect sizes [23].

## Results

Table 1 summarizes the demographic, physiologic, and electroencephalographic characteristics of the study group. Participants were 21 rowers (15 male, 6 female; mean age 30 ± 11 years) who finished consecutively from days 30 through 53 of the 2017 Atlantic Challenge. Twenty-four participants were recruited for qEEG, but the data of three rowers were not analyzed because of excessive qEEG artifacts or inadequate laboratory results.

**Table 1:** Demographic and electroencephalographic characteristics of the study group. Theta-beta ratio: mean theta/beta quantitative electroencephalographic (qEEG) voltage amplitude. Alpha asymmetry: percentage of qEEG recording sites exhibiting > 25% higher voltage amplitudes at the right-sided electrodes. Beta asymmetry: percentage of qEEG recording sites exhibiting > 25% higher voltage amplitudes at the left-sided electrodes. Hypoconnectivity and hyperconnectivity: inter‑hemispheric connectivity expressed as voltage amplitude coherence based on each left-right homologous electrode pair’s and each frequency band’s coherence values (0–1), Fisher‑z–transformed and standardized against an age‑matched normative database. Hypoconnectivity: percentage of pairs with z‑scores ≤ –1.96. Hyperconnectivity: percentage of pairs with z‑scores ≥ +1.96.

Characteristics	Mean ± SD
Age	30.83 ± 10.61
Sex	18 male, 6 female
Theta-beta ratio	0.51 ± 0.38
Alpha asymmetry (%)	34.58 ± 23.53
Beta asymmetry (%)	44.08 ± 14.11
Hypoconnectivity (%)	1.42 ± 3.49
Hyperconnectivity (%)	90.75 ± 13.33

Table 2 summarizes the pre- vs. post-race changes in anthropomorphic characteristics and the physiological, stress-related biomarkers of the study participants.

**Table 2:** Anthropomorphic and physiologic stress-related biomarkers. Weight: body weight, Waist: waist circumference, Fat: body fat percentage, Test: testosterone, MYO: myoglobin, TAC: total antioxidant capacity, MDA: malondialdehyde oxidative stress marker, COMP: collagen oligomeric matrix protein, IL-6: interleukin-6, BKA: bacterial killing activity, HCA: hemolytic complement activity, p-value: paired two-tailed t-test significance, NS: not significant. Values are rounded to the nearest whole number for readability.

Variables	Pre-race	Post-race	p-value
Weight (kg)	± 15	± 13	< 0.0001
Waist circumference (cm)	87 ± 9	81 ± 7	< 0.0001
Fat (%)	18 ± 4	13 ± 4	< 0.0001
Leptin (ng/mL)	5 ± 3	2 ± 2	< 0.0001
TEST (pg/mL)	60 ± 44	52 ± 37	> 0.1 NS
Cortisol (ug/dL)	0.2 ± 0.1	0.3 ± 0.2	< 0.05 (one-tailed)
MYO (ng/mL)	305 ± 438	570 ± 427	< 0.001
TAC (mM)	0.8 ± 0.2	0.8 ± 0.3	> 0.1 NS
MDA (pg/mL)	115 ± 54	122 ± 67	> 0.1 NS
COMP (pg/mL)	2066 ± 1370	2362 ± 1915	> 0.1 NS
IL-6 (pg/mL)	2 ± 2	6 ± 11	< 0.05 (one-tailed)
BKA (%)	0.7 ± 0.3	0.7 ± 0.3	> 0.1 NS
HCA (CH_50_)	0.03 ± 0.00	0.02 ± 0.01	> 0.1 NS

As expected, the anthropomorphic markers of body weight, waist circumference, and body fat percentage decreased. The physiological stress marker cortisol increased. The energy metabolism marker plasma leptin decreased, and both the muscle breakdown marker myoglobin and the immunologic marker IL-6 increased. Prior studies consistently report significant increases in cortisol and IL-6 associated with exercise stress [20]. These findings provided the rationale for using one-tailed, rather than two-tailed, t-tests to evaluate the significance of changes in these markers.

A greater loss in body weight from pre- to post-race was associated with lower increases in physiological stress, as indicated by a negative correlation with plasma cortisol levels (r = -0.48, p < 0.05), and with lower musculoskeletal stress, as reflected by a negative correlation with plasma COMP levels (r = -0.4, p < 0.05). This suggests that rowers who maintained more of their body weight experienced higher stress responses, potentially due to reduced fluid loss or metabolic adaptations.

A greater percentage loss in body weight was strongly associated with changes in hormonal energy balance, as indicated by a positive correlation with leptin levels (r = +0.62, p < 0.01). Specifically, rowers who lost more body mass tended to experience a greater increase in physiological (cortisol) and musculoskeletal (COMP) stress markers, while showing a greater decline in leptin, suggesting a shift in energy regulation in response to race demands.

Significant correlations were found between the percent changes in innate immune response markers (BKA and HCA) from pre- to post-race and post-race qEEG beta voltage amplitudes (R² > 0.16, p < 0.05). Notably, these were the only biomarkers among those studied that showed a significant relationship with any qEEG parameter, suggesting a potential link between immune system activation and neural activity following the transatlantic rowing race.

**Figure 1:**
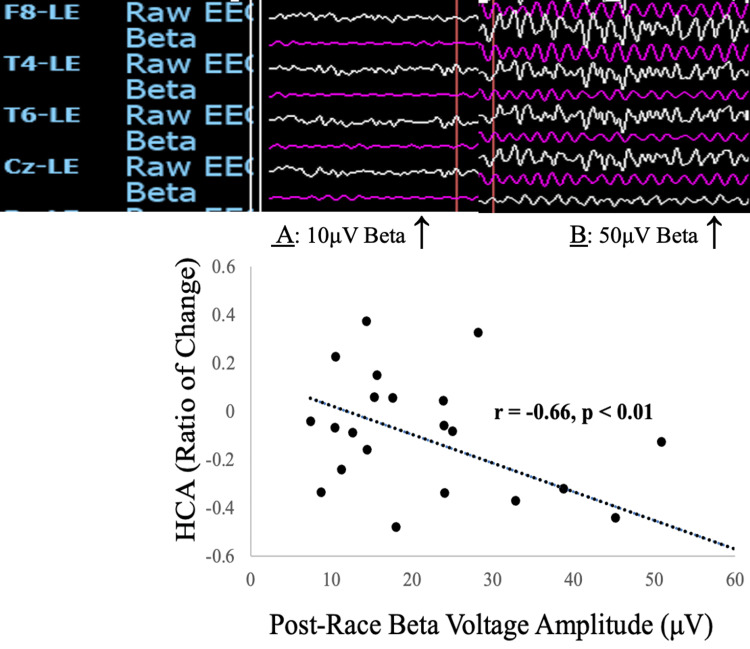
Relationship between beta brainwave and hemolytic complement activity. Linear regression trendline demonstrating the relationship between post-race quantitative electroencephalographic (qEEG) beta (13–30 Hz) brainwave voltage amplitudes (μV), measured under eyes-closed conditions, and the pre- versus post-race ratio of change for a hemolytic complement activity (HCA) assay. Participants were 21 rowers (15 male, 6 female) studied at the finish line of a 3,000-mile transatlantic rowing competition. Pearson’s correlation coefficient: –0.66, significant at p < 0.01. Arrows point to representative raw qEEG (white tracing) and filtered beta wave qEEG (pink tracing) from participant A (mean beta voltage amplitude: 10 μV) and participant B (mean beta voltage amplitude: 50 μV), representing the lowest and highest post-race beta voltage amplitudes, respectively. Statistical power: 0.92.

Figure 1 shows that the qEEG beta voltage amplitudes, measured under eyes-closed conditions, were negatively correlated with the pre- versus post-race percent changes in hemolytic complement activity (HCA, r = -0.66, p < 0.01).

The post-race qEEG beta voltage amplitudes measured under eyes-open conditions were also negatively correlated with the pre- versus post-race ratios of change in HCA (r = -0.65, p < 0.01; statistical power = 0.91).

The qEEG beta voltage amplitudes, measured under eyes-closed and eyes-open conditions, were positively correlated with the pre- versus post-race ratio of changes in BKA (r = +0.44, +0.44, p < 0.05).

Correlations between the pre- versus post-race changes in BKA or HCA and the post-race qEEG delta, theta, and alpha voltage amplitudes were not significant (R² < 0.16, p > 0.1). Significant correlations between the pre- versus post-race changes in the biomarkers and the qEEG dominant (mode) frequencies and coherences were not found for the four bandwidths studied: delta, theta, alpha, and beta. Correlations between the pre- versus post-race changes in BKA or HCA and the post-race qEEG dominant (mode) frequencies or the post-race qEEG coherences were also not significant for the four bandwidths studied: delta, theta, alpha, and beta.

## Discussion

The main findings of this study are that increased voltage amplitudes of fast beta waves, measured under both eyes-open and eyes-closed conditions, were negatively correlated with pre- to post-race decrements in HCA and were positively correlated with pre- versus post-race enhancement of BKA. The statistical power of the HCA finding was more than adequate (>0.9), although that of the BKA finding was underpowered (<0.8). The correlations were based on the ratio of change using the post-race values rather than the baseline values as the reference point. We employed this method for computing the pre- versus post-race changes to enable a more physiologically coherent comparison of neural activity under conditions of maximal exertional stress or acute recovery. This approach limits the variability in baseline factors such as training level, hydration, nutrition, and anticipatory stress. It also facilitates a more direct and interpretable correlation between end-point brain activity and systemic adaptations occurring across the stressor interval. These results appear to reflect a significant correlation between the beta voltage amplitude and two measures of adaptive modulation of the innate immune system in response to the extreme physical stress of a transatlantic rowing competition covering approximately 3,000 miles in 30 to 53 days.

The present study builds upon findings from a pilot case series reinforcing the hypothesis that prolonged intense physical exertion induces neurophysiological adaptations [14]. The association between fast beta wave activity and immune modulation (increased BKA and decreased HCA) raises the possibility that cortical arousal may be involved in coordinating immune responses during the extreme stress of transoceanic rowing; however, this interpretation remains speculative and requires validation through longitudinal and mechanistic studies. Compared to the pilot cohort, the larger sample in this study provides evidence for the association between beta wave dominance and heightened vigilance. The increased beta coherence observed post-race suggested a neuroadaptive mechanism to conserve energy during prolonged stress, aligning with theories of neural efficiency [14]. These findings corroborate earlier studies linking beta wave activity with stress-induced arousal and cognitive task performance under demanding conditions. The shifts in alpha asymmetry observed in the pilot study were further validated, with left-sided dominance potentially reflecting enhanced parasympathetic activity and oxidative stress adaptation. This mechanism may support the transition to a recovery state, optimizing energy conservation while maintaining readiness for future stressors.

Results of the present study suggest that there may be reallocation of metabolic energy to maintain a state of high vigilance and innate immune responsiveness during the extreme stress of a transatlantic rowing competition. Higher beta voltage amplitudes were significantly associated with high BKA and with low HCA. This observed pattern of higher BKA alongside lower HCA under extreme, prolonged stress may suggest a shift in innate immune function; however, further longitudinal studies are needed to determine whether this reflects an adaptive response [24, 25]. Bacterial killing assays assess the direct elimination of pathogens, which is critical in combating infections, particularly in high-stress environments where exposure to pathogens may be elevated. In contrast, HCA, while vital for classical pathway-mediated immune responses, is energetically costly and may involve non-specific tissue damage due to excessive complement activation. Evolutionarily, bacterial killing mechanisms, which include antimicrobial peptides and phagocytosis, emerged early in metazoan development and represent a conserved, frontline defense mechanism crucial for survival in variable environments [26]. Conversely, the complement system, while critical, is a later evolutionary development with roles extending beyond pathogen clearance, including immune regulation and inflammation, which may be less immediately essential during stress [27]. Embryologically, bacterial killing mechanisms are functional early in life, while full complement activity matures later, reflecting their relative urgency in early survival versus long-term immune balance [28]. This evolutionary and developmental divergence suggests that, during prolonged stress, the innate immune system may prioritize bacterial killing, an energetically efficient and evolutionarily ancient response, over complement-mediated hemolysis, aligning with the body's need to conserve resources and minimize collateral damage.

Brain wave adaptations in ultra-endurance studies

Other ultra-endurance EEG studies have reported quite different spectral shifts than the post-race beta dominance seen in our rowers. For example, a 330-km mountain ultramarathon is reported to increase the voltage amplitude of very slow bandwidth waves (delta and theta) and decrease that of the posterior alpha, without any significant change in the beta-band activity [29]. The voltage amplitude power is reported to increase in all the EEG bandwidths during an early, low-fatigue phase while running a marathon, associated with a greater percentage increase in alpha compared to beta power [30]. Decrements in frontal alpha and delta power are reported, also in the absence of changes in beta power, in a resting EEG study of 24 marathoners several days post-race [31]. In contrast, our transoceanic rowers appeared to show a post-race extreme elevation of beta voltage amplitude, evidenced by extremely low theta-beta ratios, averaging 0.5 compared to normal values of 2.0 to 4.0. Although we could not measure their pre-race values, it is highly unlikely that their theta-beta ratios would have been that low. Although preliminary, this finding suggests that the continuous cognitive demands and sleep deprivation induce a distinct arousal-vigilance signature in the qEEG that may be unique to the rigor of a transoceanic multi-week race.

Immune adaptations in ultra-endurance studies

The immunologic adaptations in other endurance activities show both parallels and contrasts to the present report. Extreme exercise generally mobilizes innate defenses, but the balance between bacterial killing and complement activation can vary by event. For example, participants in a 100-km ultramarathon had significant increases in both functional bacterial killing ability and HCA from pre- to post-race [24, 25]. This finding is consistent with the notion that acute stress upregulates broad innate responses (e.g., neutrophil activity, antimicrobial proteins). In our rowers, however, complement activity actually decreased, even as bacterial killing increased. One interpretation is that extremely prolonged stress leads to a strategic reallocation: energetically costly complement-mediated inflammation is downregulated while direct antibacterial defenses are prioritized. Importantly, the increase in bacterial-killing capacity, in and of itself, aligns with the general finding that ultra-endurance augments immediate pathogen defense. Our results, therefore, appear to complement existing reports by showing that while ultra-endurance races tend to amplify innate immunity, the specific pattern of immune effectors (BKA vs. HCA) may shift with the duration and nature of the event.

Metabolic adaptations in ultra-endurance studies

The metabolic and hormonal stress responses in our rowers likewise mirror known patterns from other endurance competitions. Sustained exercise of this magnitude consistently induces substantial energy deficits, weight loss, and catabolic endocrine changes. In the 100-km runners studied by Longman and associates, the athletes lost on average approximately 1.8 kg and exhibited an increase in cortisol combined with a decrease in testosterone, reflected in a decreased T/C ratio [24, 25]. A similar endocrine profile, wherein post-race cortisol levels increased while anabolic steroids decreased, is reported for participants in Ironman competitions [32]. These shifts correspond with substrate utilization changes. In one marathon case study, the respiratory exchange ratio decreased from approximately 1.0 toward 0.83 in the latter stages, indicating a switch from carbohydrate to fat metabolism as glycogen became depleted [30]. In the present study, the transatlantic rowers almost certainly underwent a comparable energy reallocation. These comparisons suggest that very long duration endurance produces a high-stress metabolic state characterized by elevated stress hormones, suppressed sex steroids, and conserved energy metabolism, consistent with the resource reallocation and survival prioritization we infer from our rowing cohort.

Limitations and future directions

This article is intended as a preliminary report. The lack of pre-race EEG baselines and the correlational design limit the statistical power and strength of causal or evolutionary interpretations, which await further clarification by a larger study. In the absence of a control group, pre-race qEEG, and blinding, this article reports the results of a preliminary study, the data of which should be interpreted cautiously pending a more complete study based on recruitment of a larger number of subjects. While the study provides compelling evidence for significant associations between qEEG signatures, metabolic markers, and immune function, its cross-sectional design limits our ability to make inferences relevant to cause-effect relationships. Future longitudinal studies with pre- and post-race measurements are necessary to delineate the temporal sequence of these changes. Additionally, integrating more diverse biomarkers, such as oxidative stress indicators and inflammatory cytokines, will further elucidate the mechanistic pathways underlying these neurophysiological adaptations.

## Conclusions

The results of the present study align with the hypothesis that extremely high beta brainwave activity is associated with systemic metabolic and immune changes during the extreme stress of transoceanic rowing. This investigation underscores the value of qEEG as a noninvasive tool for studying stress adaptations in ultra-endurance athletes. It also highlights the potential for applying these findings to other contexts, such as military training, space exploration, and high-stress occupations, where understanding and enhancing resilience to extreme stressors are needed for the successful completion of critical tasks or missions.
