# Correction: Waves of Endurance: A Pilot Study of Beta Brain Wave Signatures Linked to Immune Adaptation in Transatlantic Rowers

**DOI:** 10.7759/cureus.c226

**Published:** 2025-05-29

**Authors:** Merin Chandanathil, Daniel P Longman, Tomasz Nowak, Jonathan C.K. Wells, Jay T Stock, Michael P Muehlenbein, Hannah Schneiders, Nicola L Kelly, Vasavi R Gorantla, Courtney C Lewis, Richard M Millis

**Affiliations:** 1 Physiology, American University of Antigua, St. Johns, ATG; 2 School of Sport, Exercise and Health Sciences, Loughborough University, Loughborough, GBR; 3 Anthropology, Baylor University, Waco, USA; 4 Childhood Nutrition Research Centre, UCL (University College London) Institute of Child Health, London, GBR; 5 Anthropology, University of Western Ontario, London, CAN; 6 Department of Anthropology, Baylor University, Waco, USA; 7 Anesthesiology, Southeast Scotland School of Anaesthesia, Edinburgh, GBR; 8 Medicine, University of Exeter and Torbay and South Devon NHS Foundation Trust, Torquay, GBR; 9 Medical Education, California University of Science and Medicine, Colton, USA; 10 Clinical Medicine, American University of Antigua, St. Johns, ATG

This article has been corrected to include Michael P. Muehlenbein in the author list after he was erroneously omitted during the initial submission.

